# Hospital-Owned Apps in Taiwan: Nationwide Survey

**DOI:** 10.2196/mhealth.8636

**Published:** 2018-01-16

**Authors:** Hao-Yen Liu, Wui-Chiang Lee, Ying-Chou Sun, Jun-Jeng Fen, Tzeng-Ji Chen, Li-Fang Chou, Shinn-Jang Hwang

**Affiliations:** ^1^ Department of Family Medicine, Taipei Veterans General Hospital Taipei Taiwan; ^2^ Department of Medical Affairs and Planning, Taipei Veterans General Hospital Taipei Taiwan; ^3^ School of Medicine, National Yang-Ming University Taipei Taiwan; ^4^ Department of Radiology, Taipei Veterans General Hospital Taipei Taiwan; ^5^ Department of Information Management, Taipei Veterans General Hospital Taipei Taiwan; ^6^ Department of Public Finance, National Chengchi University Taipei Taiwan

**Keywords:** hospitals, telemedicine, mobile apps, Taiwan, mHealth

## Abstract

**Background:**

Over the last decade, the use of mobile phone apps in the health care industry has grown rapidly. Owing to the high penetration rate of Internet use in Taiwan, hospitals are eager to provide their own apps to improve the accessibility of medical care for patients.

**Objective:**

The aims of this study were to provide an overview of the currently available hospital-owned apps in Taiwan and to conduct a cross-hospital comparison of app features.

**Methods:**

In May 2017, the availability of apps from all 414 hospitals in Taiwan was surveyed from the hospital home pages and the Google Play app store. The features of the downloaded apps were then examined in detail and, for each app, the release date of the last update, download frequency, and rating score were obtained from Google Play.

**Results:**

Among all the 414 hospitals in Taiwan, 150 (36.2%) owned Android apps that had been made available for public use, including 95% (18/19) of the academic medical centers, 77% (63/82) of the regional hospitals, and 22.0% (69/313) of the local community hospitals. Among the 13 different functionalities made available by the various hospital-owned apps, the most common were the doctor search (100%, 150/150), real-time queue monitoring (100%, 150/150), and online appointment scheduling (94.7%, 142/150) functionalities. The majority of apps (57.3%, 86/150) had a rating greater than 4 out of 5, 49.3% (74/150) had been updated at some point in 2017, and 36.0% (54/150) had been downloaded 10,000 to 50,000 times.

**Conclusions:**

More than one-third of the hospitals owned apps intended to increase patient access to health care. The most common app features might reflect the health care situation in Taiwan, where the overcrowded outpatient departments of hospitals operate in an open-access mode without any strict referral system. Further research should focus on the effectiveness and safety of these apps.

## Introduction

### Access to Mobile Health

Over the past few years, the proportion of the global population with access to mobile phone technology has surged, and it will continue to increase further, rising from an estimated 36.7% in 2016 to 74.7% in 2021 [1]. Mobile technology has penetrated every aspect of daily life, including health care. Mobile health (mHealth) apps are software tools that provide users with access to health-related information, facilitate health management, and provide connections between health care providers and users. At present, there are an estimated 259,000 mHealth apps available, from a total of 58,000 mHealth publishers, on the two main mobile device operating systems [2], and the global market for mHealth technology is expected to grow at an annual rate of 28.6% [3].

Thus far, mHealth apps have been shown to improve health outcomes by, for example, improving patients’ medication adherence and adherence to lifestyle modifications [4-6], enhancing the control of risk factors for chronic diseases [7], changing health behaviors relating to smoking cessation [8], or providing access to health care professionals for the purposes of clinical decision making and patient monitoring [9]. For health providers, previous studies have shown that mHealth interventions are cost-effective and/or cost saving [10]. Some of the mHealth apps developed by hospitals are designed to enhance patient access, to improve communications between patients and providers, or to establish the hospital’s brand online. One report in 2015 found that 66 of the 100 largest hospitals in the United States have their own mHealth apps, but that only 2% of the patients seen by those hospitals currently use those apps [11]. A US survey in 2016 claimed 52% of hospitals currently use three or more connected health technologies including mobile apps for patient education or engagement [12]. In China, Internet hospitals provide innovative health care directly through Internet technologies such as websites and mobile apps. In a study of the 43 established Internet hospitals, 18 (42%) of the hospitals provide access to outpatient health care via mobile app [13]. Related studies have focused on the contributions from the use of mHealth apps to specific disease outcomes [14-16]. However, to the best of our knowledge there have been no previous studies, much less any nationwide studies, that have analyzed the features of the mHealth apps provided by hospitals.

### The mHealth System in Taiwan

Taiwan ranks fifth among the nations of the world in terms of mobile phone penetration, with approximately 70.4% of the population owning a mobile phone [17]. When it comes to the availability of a 3G or better data signal, Taiwan is among the top 10 nations worldwide, with an availability rate of 93.87% [18]. According to a recent report, Google’s Android system dominates the market in Taiwan, being the operating system used on 68% of mobile devices [19]. Under Taiwan’s National Health Insurance (NHI) system, citizens are granted free access to any specialist, even without a referral [20]. There are multiple ways to schedule a health care appointment in Taiwan, including by phone, online, or by showing up in person. Accordingly, it has become increasingly common for hospitals to ensure their patient volumes by releasing their own mHealth apps allowing for appointment scheduling, among other features.

The aim of this study was to provide an overview of hospital-owned apps in Taiwan. To do so, we recorded basic information about these hospital-owned apps and analyzed their various features to understand their value to health care consumers, which may in turn reflect the current health care situation in Taiwan. The findings of the study may thus provide guidance to health care providers as they seek to improve their mobile strategies and expand their service offerings.

## Methods

### Data Collection

A total of 416 hospitals in Taiwan received government accreditation from 2012 to 2015 [21]. Hospitals are accredited by the Taiwan Joint Commission on Hospital Accreditation, which is supervised by the Ministry of Health and Welfare, and classified into three levels based on health care quality, medical teaching ability, clinical capabilities, and bed capacity. In our study, the three levels of the surveyed hospitals were academic medical centers, regional hospitals, and local community hospitals.

The locations of the hospitals were categorized according to the urbanization stratification of Taiwan’s 368 townships developed by Taiwan’s National Health Research Institutes [22]. Of the seven urbanization levels in that stratification, we defined levels 1 and 2 as urban, levels 3 and 4 as suburban, and levels 5 to 7 plus the isolated islands as rural. We excluded two hospitals located on remote islands because they are not included in the 368 townships.

Because of the higher prevalence of Android phone use in Taiwan and because information regarding the number of downloads was not available for iOS apps, we limited our study to Android system apps only. We used the name of each hospital to conduct a search for the given hospital’s apps in the Google Play app store (Google Inc, Mountain View, CA, USA). We also created a user account and downloaded all the hospital-owned apps to a single Android phone (HTC One X9, HTC Corporation, Taoyuan, Taiwan) in May 2017. Apps were excluded if they were not available at that time or if the given app had no features relevant to health care availability for consumers. For the 414 hospitals investigated, our searches of the Google Play store identified a total of 150 apps.

### Parameters of Consumer Interactions

We recorded basic data on the total number of reviews for all versions of each app using the data that was publically available via Google Play as of May 2017. Thus, the data analyzed was cross-sectional in nature. The average user ratings for the apps on a scale of one to five, categorized into three groups (4-5, 3.5-4, <3.5), were recorded. We also grouped the total number of downloads reported by Google Play into six different categories (100,000-500,000; 50,000-100,000; 10,000-50,000; 5000-10,000; 1000-5000; <1000), and recorded the given value for each app.

**Figure 1:**
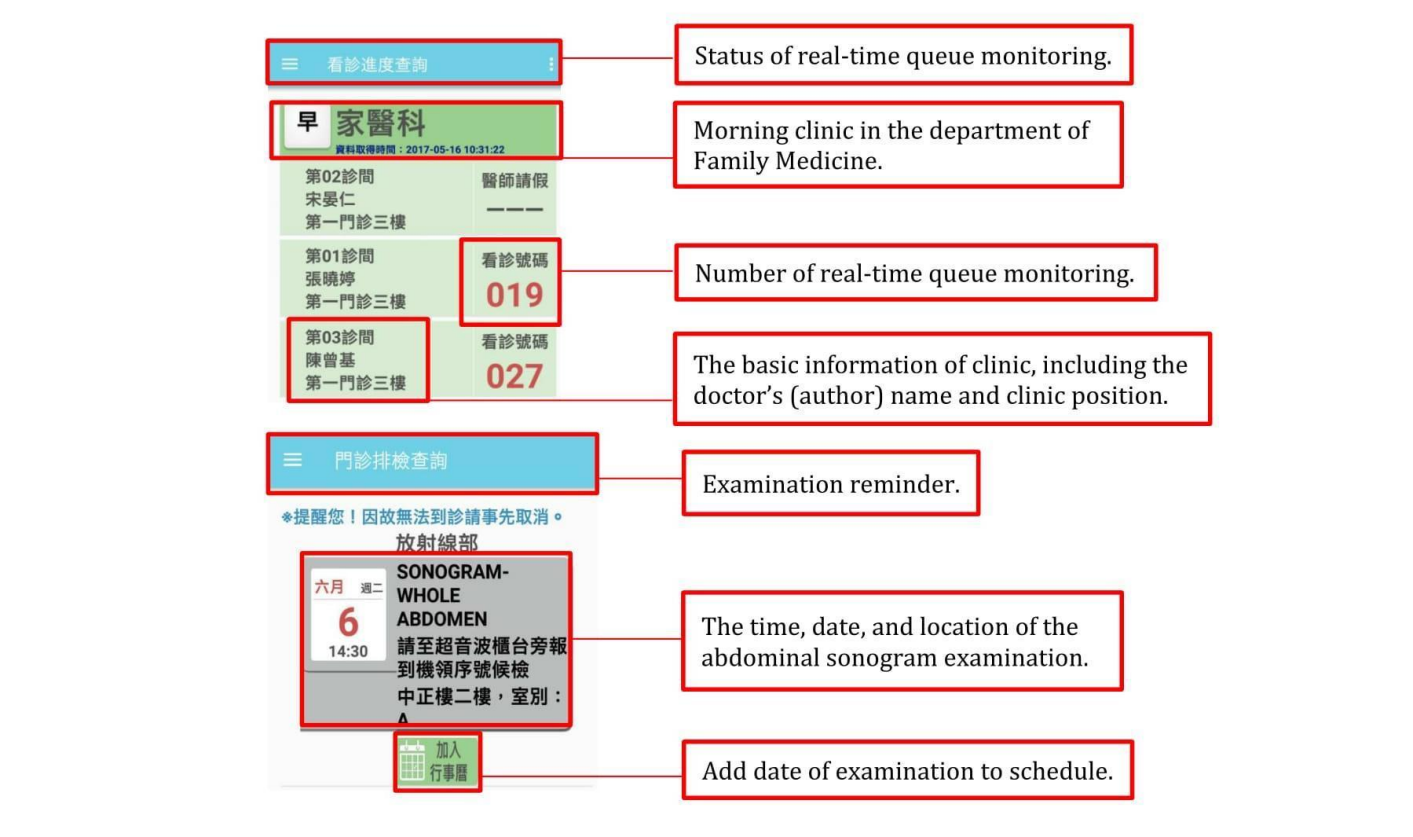
Screenshot of the real-time queue monitoring and examination reminder features of an app provided by Taipei Veterans General Hospital.

### Apps Features Extraction and Review

A Microsoft Excel worksheet was constructed for the extraction of data regarding all the included apps. We listed features of these apps that are useful when patients are seeking health care and excluded features such as advertisements, games, and location. A total of 13 features of these apps were analyzed, including real-time queue monitoring (Figure 1), doctor search, appointment scheduling, appointment reminder, mobile payment, drug information, examination report, prescription refill reminder, personal health management, satisfaction and feedback, examination schedule, parking vacancy monitoring, and multilanguage features. Two investigators independently reviewed the features of each app, and the features were then cross-verified.

### Statistical Analysis

A boxplot was constructed to present the rating scores for all the apps, which were then stratified by the three hospital types. For each boxplot of each hospital type, the bottom of the box represented the 25th percentile, the top of the box represented the 75th percentile, and the midline in the box represented the 50th percentile of the mean rating score of the app. The size of each box can provide an estimate of the rating score distribution of these apps.

## Results

### Characterization of the Hospitals

Out of the total analyzed sample of 414 hospitals, we identified 150 hospitals for which hospital-owned apps were available online, including 18 academic medical centers, 63 regional hospitals, and 69 local community hospitals. According to a 2016 report, daily outpatient visits in academic medical centers accounted for 28.81% (106,458/369,552) of the total hospital outpatient visits, followed by 30.79% (113,785/369,552) in regional hospitals and 34.07% (125,913/369,552) in local community hospitals [23]. All these hospitals provided Internet-based appointment scheduling systems through their home pages. Over the course of this study, all the Android apps developed by these hospitals appeared in the search results for the names of the different hospitals. All the academic medical centers (n=18) were located in urban regions. Of the regional hospitals (n=63), 68% (43/63) were located in urban regions, 30% (19/63) were located in suburban regions, and 2% (1/63) were located in rural regions. Of the local community hospitals (n=69), 48% (33/69) were situated in urban areas, 41% (28/69) were situated in suburban areas, and 12% (8/69) were situated in rural areas.

### Distribution of Hospital-Owned Apps

As shown in Table 1, 150 of 414 hospitals had designed and made available their own apps, with those 150 including 95% (18/19) of the hospitals in the academic medical center group, 77% (63/82) of the hospitals in the regional hospital group, and 22.0% (69/313) of the hospitals in the local community hospital group. These results indicated notable differences among the three different types of hospitals. However, the proportions of hospitals with their own apps did not differ in terms of the three different urbanization levels.

**Table 1:** Proportion of apps (n=150) developed in all hospitals (N=414), stratified by accreditation type and urbanization level^a^.

Hospital characteristics	Academic medical center (n=19), n/N (%)	Regional hospital (n=82), n/N (%)	Local community hospital (n=313), n/N (%)	Total (N=414), n/N (%)
Urban (n=253)	18/19 (95)	43/56 (77)	33/178 (18.5)	94/253 (37.2)
Suburban (n=135)	N/A^b^	19/25 (76)	28/110 (25.5)	48/135 (35.6)
Rural (n=26)	N/A^b^	1/1 (100)	8/25 (32)	9/26 (35)
Total (N=414)	18/19 (95)	63/82 (77)	69/313 (22.0)	150/414 (36.2)

^a^Values are hospitals with apps/all hospitals (percentages) unless otherwise indicated.

^b^N/A: not applicable.

**Table 2:** Content and features of hospital-owned apps (N=150).

Apps characteristics		Academic medical center (n=18), n (%)	Regional hospital (n=63), n (%)	Local community hospital (n=69), n (%)	Total (N=150), n (%)
**Last update time**					
	2017	10 (56)	28 (44)	36 (52)	74 (49.3)
	2016	6 (33)	20 (32)	22 (32)	48 (32.0)
	2015 and before	2 (11)	15 (24)	11 (16)	28 (18.7)
**Features**					
	Real-time queue monitoring	18 (100)	63 (100)	69 (100)	150 (100)
	Doctor search	18 (100)	63 (100)	69 (100)	150 (100)
	Appointment scheduling	18 (100)	59 (94)	65 (94)	142 (94.7)
	Appointment reminder	5 (28)	20 (32)	17 (25)	42 (28.0)
	Mobile payment	11 (61)	11 (17)	12 (17)	34 (22.7)
	Drug information	8 (44)	14 (22)	12 (17)	34 (22.7)
	Examination report	7 (39)	11 (17)	14 (20)	32 (21.3)
	Prescription refill reminder	6 (33)	9 (14)	10 (14)	25 (16.7)
	Personal health management	3 (17)	9 (14)	10 (14)	22 (14.7)
	Satisfaction and feedback	3 (17)	3 (5)	9 (13)	15 (10.0)
	Examination schedule	1 (6)	1 (2)	1 (1)	3 (2.0)
	Parking vacancy monitoring	2 (11)	N/A^a^	1 (1)	3 (2.0)
	Multilanguage	1 (6)	1 (2)	1 (1)	3 (2.0)

^a^N/A: not applicable.

### Features of Apps

Of the 150 apps, all had real-time queue monitoring and doctor search capabilities. In terms of availability, those capabilities were followed by appointment scheduling (94.7%, 142/150), appointment reminder (28.0%, 42/150), drug information (22.7%, 34/150), mobile payment (22.7%, 34/150), prescription refill reminder (16.7%, 25/150), personal health management (14.7%, 22/150), and satisfaction and feedback (10.0%, 15/150) functions. Only three of the 150 apps offered content in multiple languages, including English, as well as parking vacancy monitoring and examination reminder functions. Table 2 summarizes the distribution of the 13 app features across the different hospital levels.

**Figure 2:**
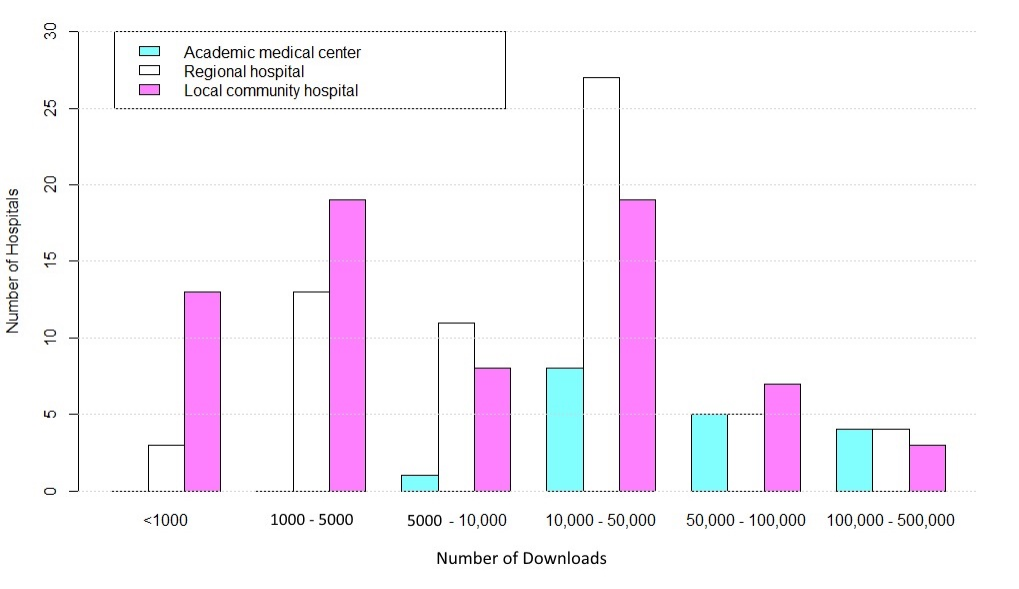
Download distribution of hospital-owned apps (N=150).

For the number of app downloads, the apps for 36.0% (54/150) of the hospitals had been downloaded 5000 to 10,000 times (Figure 2). Among the academic medical centers, half (9/18) of the corresponding apps had been downloaded 100,000 to 500,000 or 50,000 to 100,000 times. In contrast, 46% (32/69) of the local community hospital apps were downloaded fewer than 5000 times. Regarding the date of the most recent update, 49.3% (74/150) of the hospitals with apps had updated their respective apps in 2017. There were no significant differences in update date among the different hospital accreditation levels.

**Figure 3:**
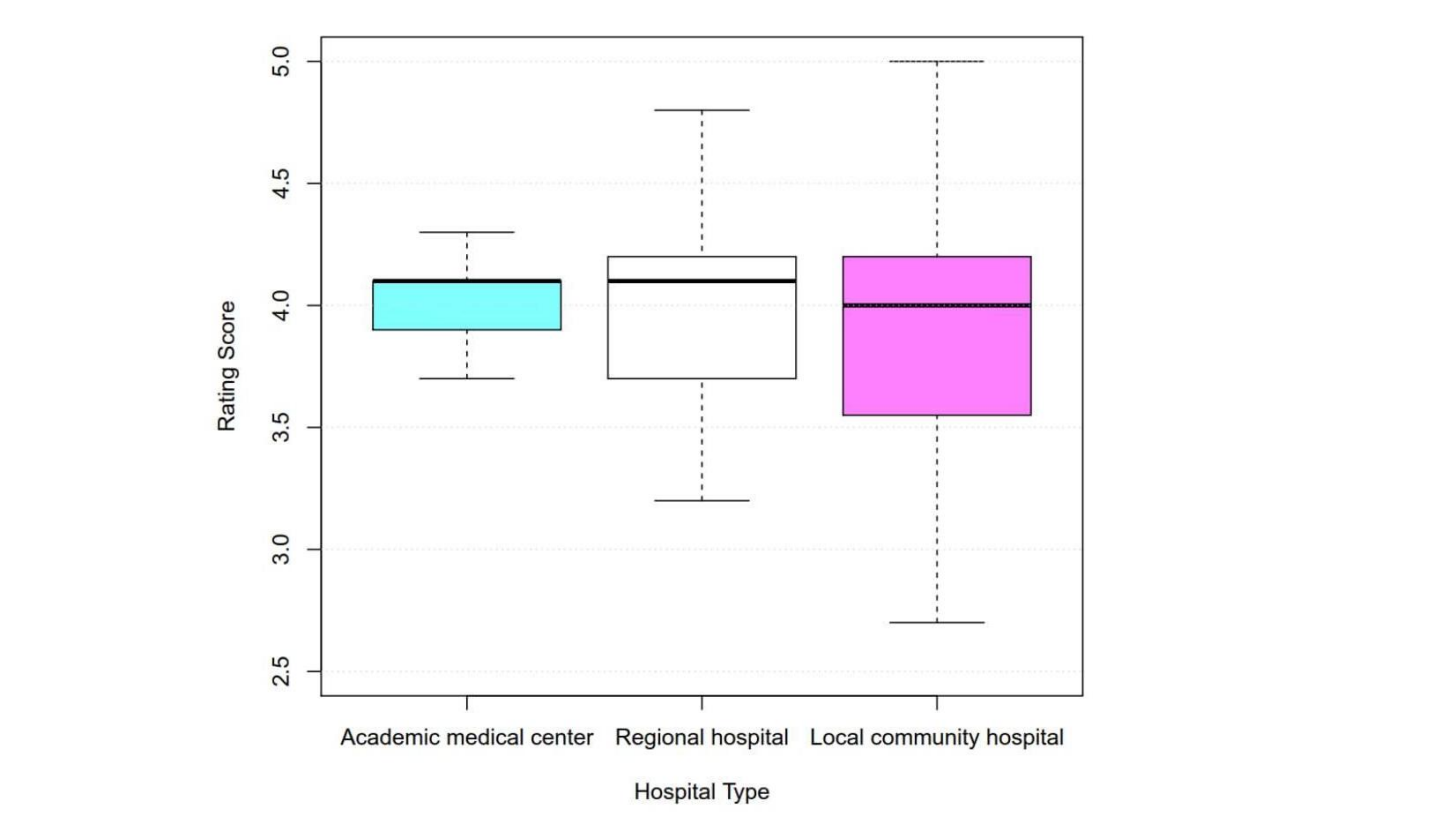
Users’ rating scores by hospital type.

The majority of the apps (57.3%, 86/150) had received a high mean rating of between 4 and 5, 27.3% (41/150) had received a mean rating of between 3.5 and 4, and 14.0% (21/150) had received a poor mean rating (ie, mean rating <3.5). We also used the rating score data to illustrate the rating score distributions for the various apps according to the three different types of hospitals (Figure 3). The mean rating scores were 4.01 (SD 0.21) for the academic medical center apps, mean 3.99 (SD 0.42) for the regional hospital apps, and mean 3.89 (SD 0.70) for the local community hospital apps. The box in Figure 3 was largest for the local community hospital group apps, indicating that the ratings for those apps exhibited the largest degree of variation, followed by the box for the regional hospital group apps and the box for the academic medical center group apps.

## Discussion

### Principal Findings

The results of this study provide an overview of the distribution of hospital-owned apps in Taiwan as of May 2017. The differences in said distribution among the three types of hospitals and among the hospitals in the different types of regions might reflect the demands of the patients and the mobile penetration rates for the hospitals of the different types and regions. The key features of the identified mHealth apps were appointment scheduling, real-time queue monitoring, and doctor search functions, followed by appointment reminder, drug information, mobile payment, personal health management, satisfaction and feedback, and other functions. Approximately half (49.3%, 74/150) of the apps were updated in 2017, 36.0% (54/150) had been downloaded 10,000 to 50,000 times, and 57.3% (86/150) had a rating greater than 4 out of 5. All the available apps could be downloaded for free.

### Distribution of Apps

This study found that the proportion of hospital-owned apps was significantly higher among hospitals of larger scale. The citizens of Taiwan can visit any level of hospital directly without a referral [20]. According to the 2015 annual report from the Ministry of Health and Welfare, the proportion of patient encounters in all hospitals was 36% (n=402,621 per day; 10.4%, 13.3%, and 9.3% in academic medical centers, regional hospitals, and local community hospitals, respectively) in 2015 [24,25]. Owing to greater financial, research, and educational resources, academic medical centers were often the preferred alternative for citizens in Taiwan, even though they typically charge higher co-payments and are more likely to be overcrowded. To remain competitive and ensure the maintenance of their incomes, these hospitals have aggressively increased access to care, leading to increased market share. In this survey, almost all the academic medical centers had made their own apps available, with the exception of one with an Internet-based appointment service only. Among the local community hospitals, fewer had their own apps and of those that did have apps, half of them had a number of downloads less than 5000. A possible reason for this might be the relatively low service volumes of local community hospitals compared to academic medical centers and regional hospitals. It was notable that we found similar proportions of hospitals with their own apps among regions with different urbanization levels. As such, whether there is a significant digital divide between hospitals in urban and rural areas requires further research.

### Features of Apps

#### Appointment Reminder

Overall, nearly all the apps had the core features of real-time queue monitoring, appointment scheduling, and doctor search. In Taiwan, the average outpatient department visit rate has been reported to be up to 14 times per year per person, higher than the rates in other countries [20]. In the overcrowded outpatient clinics, which generally do not maintain waiting lists, these high-yield features of apps are particularly desired by patients seeking better access to appointments and the ability to monitor clinic queues regardless of time and place. More than one in four apps in the study also had an appointment reminder feature. Previous studies have reported that appointment reminder systems, such as short message service text messaging, can increase attendance at appointments and improve cancelation and rescheduling of unwanted appointments [26,27]. In addition, mHealth reminder systems have been reported to improve no-show rates and be more cost-effective than conventional strategies [28,29].

#### Medication Adherence

Poor medication adherence is particularly problematic among patients with chronic illnesses, and increasing adherence might have greater impact on health outcomes than specific medical therapies [30,31]. Owing to the large number of patients that they see, physicians in Taiwan usually spend less time on health counseling, including education regarding medication usage, than doctors in other countries [32]. In this study, we noticed that the prescription refill reminder and drug information features of apps were mainly aimed at improving patients’ medication adherence and self-management. However, there is still a lack of evidence that mobile apps have a beneficial effect on medication adherence [33,34].

#### Mobile Payment

Mobile payment technology has gained widespread adoption in the restaurant and retail industries, but most health care providers still receive paper-based payments from patients. However, a report in 2016 found that mobile payments had doubled to 20% of all online payments in the preceding 3 years, and that consumer demand for convenience through online payment channels was increasing [35]. Accordingly, hospitals have begun to integrate mobile payment systems into their mHealth apps. In this study, approximately one-fifth of the identified apps provided users options for paying their medical bills via the digital platform. As such, instead of waiting in front of a clerk, patients can reduce unnecessary wait times in hospitals. For health care providers, such mobile payment options might give staff members more flexibility and availability by reducing the associated paper work.

#### Personal Health Record and Safety Concern

In this study, approximately one-fifth of the identified apps provided users with access to their examination reports and data. It is possible that patients might effectively improve their health outcomes and self-management by monitoring their personal health records via mHealth apps. On the other hand, patients might also struggle to interpret their medical data via the complex interfaces of apps [36]. Furthermore, it should also be noted that there are corresponding legal concerns and risks related to data protection [37]. For example, the personal record could be easily captured via entering the patient’s ID number without further certification from interface of some apps. We also found no sufficient information or privacy policies in the app store descriptions regarding the guidelines for restricted content or the security of the data provided via the apps. Previous studies showed that mHealth app use might make widespread use of unsecured wireless network [38]. These concerns might lead to resistance to the use of mHealth apps from stakeholders. According to one study about online health data, approximately 41% of US consumers have privacy and security concerns related to usage of mobile phone health devices [39]. In 2015, the US Food and Drug Administration released new guidance regarding the regulations for specific types of mHealth apps [40]. Increased use of mHealth apps could leave health data unsecured unless app developers keep improving the way they communicate and store data [38].

In the future, the developers of these mHealth apps should take regulation and risk assessment into consideration to ensure the safety of these apps.

### Limitations

One possible limitation of this study is that this was a cross-sectional study. All the results regarding the parameters of apps may be out of date fairly soon because existing apps are updated and additional apps are continually released. Second, the search for apps was limited to the Android app store because the Android operating system has the highest market share; therefore, the results of this study might not be representative of the apps for the iOS and other platforms. Third, we could not precisely assess the rating scores of every app because some of the apps had only received a few reviews. In addition, assessing the quality of apps in terms of the star ratings given by users may not be the most reliable method of judging said quality [41]. Future studies are thus needed to provide a multidimensional measure for the quality assessment of mHealth apps. Fourth, in our research we did not investigate how these hospital-owned apps may increase accessibility, care quality, or patient satisfaction. Further discussions will be needed especially from administrators’ points of view.

### Conclusions

More than one-third of hospitals in Taiwan had their own mHealth app aimed at increasing patient access to health care. The most common app features might reflect the current health care situation in Taiwan, where the overcrowded outpatient departments of hospitals in the NHI system operate in an open-access mode without any strict referral system. Continued research is necessary to evaluate the beneficial effects on health outcomes contributed by these mHealth apps. Developers should design features in apps that can adequately address the demands of users and should focus seriously on issues relating to the regulation and safety of these mHealth apps.

## Appendix

Multimedia Appendix 1 Proportion of apps (n=150) developed in all hospitals (N=414), stratified by accreditation type and urbanization level.a.

Multimedia Appendix 2 Content and features of hospital-owned apps (N=150).

## Abbreviations

NHI: National Health Insurance
