# Supplementary figures and images for: Hospital-Owned Apps in Taiwan: Nationwide Survey
Source: JMIR Mhealth Uhealth. 2018 Jan 16;6(1):e22. doi: 10.2196/mhealth.8636 (PMC5790962; doi:10.2196/mhealth.8636)

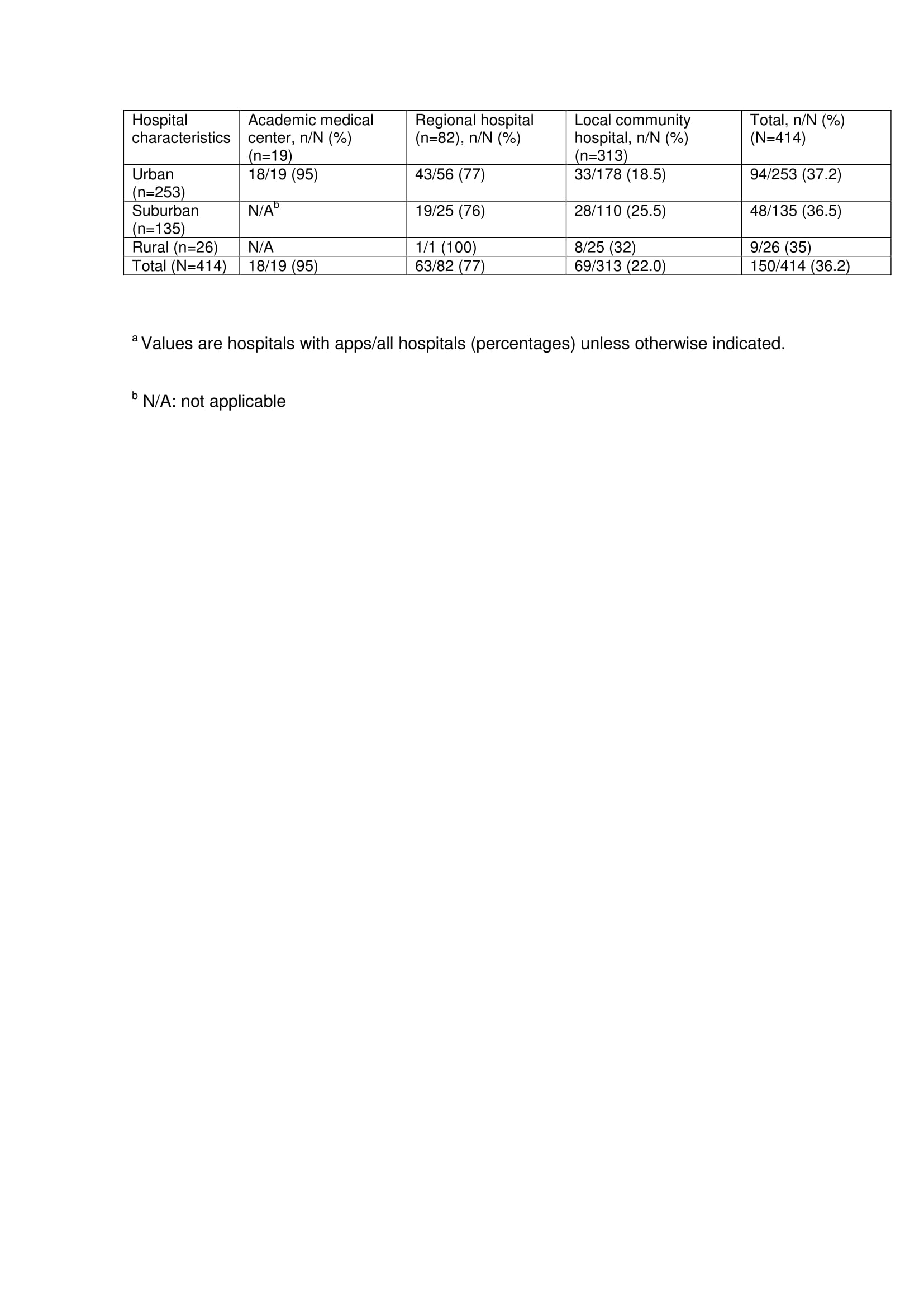

Supplement: Multimedia Appendix 1 [file mhealth_v6i1e22_app1.jpg]

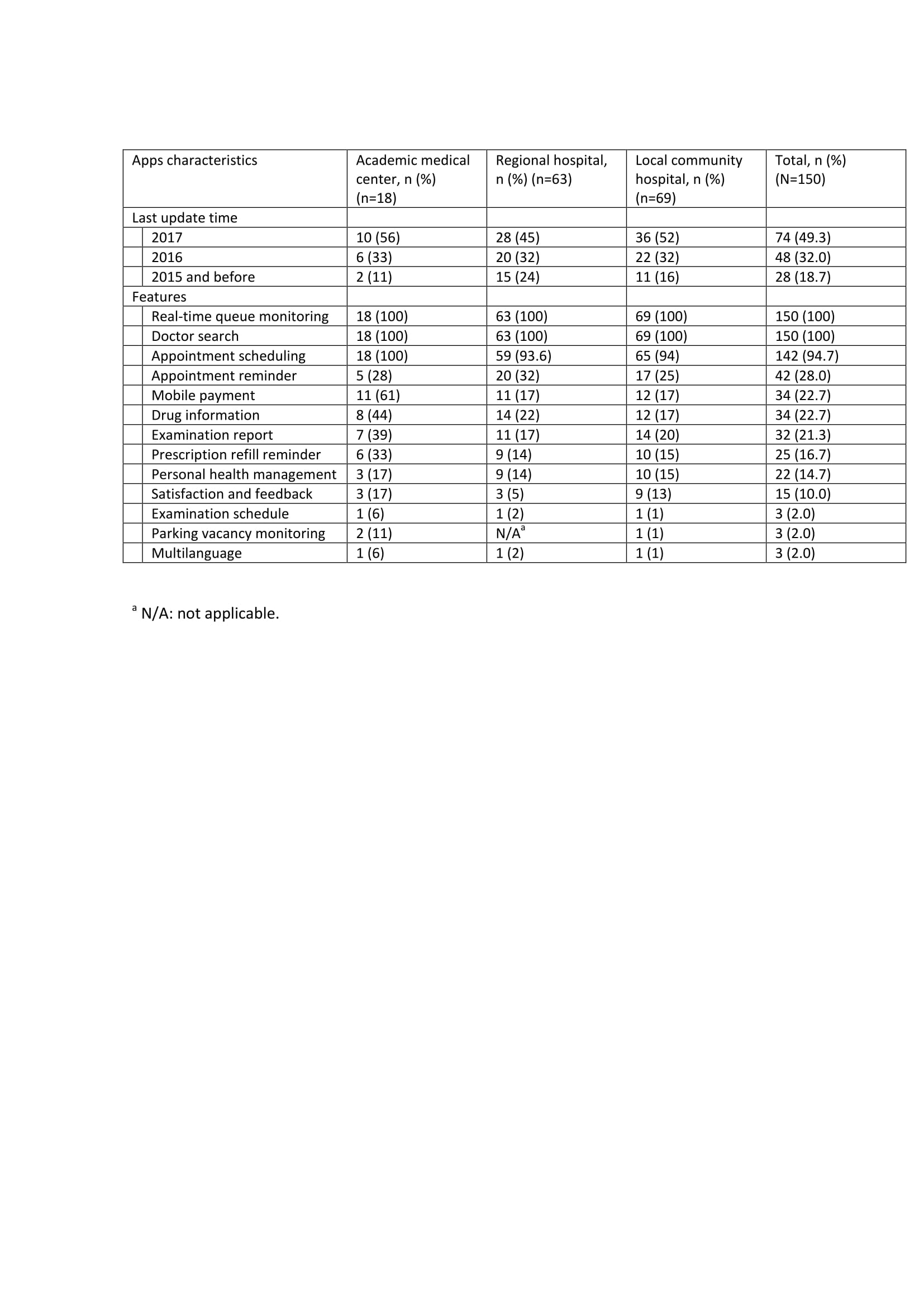

Supplement: Multimedia Appendix 2 [file mhealth_v6i1e22_app2.jpg]
